# Bioactive Materials for Next-Generation Dentistry

**DOI:** 10.3390/bioengineering9120782

**Published:** 2022-12-08

**Authors:** Gaskon Ibarretxe

**Affiliations:** Department of Cell Biology and Histology, Faculty of Medicine and Nursing, University of the Basque Country (UPV/EHU), 48940 Leioa, Spain; gaskon.ibarretxe@ehu.eus; Tel.: +34-94-601-3218

Teeth were some of the first organs whose function was effectively restored by inert refilling materials that have become widely known to the general public; amalgams, polymeric resin composites, and gutta-percha are some such examples. These materials have brought incredible benefits to the health of millions of patients worldwide. In recent decades, there has been overwhelming progress in the dental material field. Even though dental diseases like caries and periodontitis are still extremely common in humans of all ages [1], many of the practical consequences and discomfort associated with dental and periodontal decay have been largely averted thanks to modern techniques of hard and soft dental tissue restoration. However, the currently available dental refilling procedures are still far from perfect; despite their long-term stability, amalgams have progressively been abandoned because of their mercury release, risk to practitioners, and waste management issues [2,3], and the polymeric resin composites that eventually came to replace them are known to favor bacterial adherence and biofilm formation [4]. Regarding current endodontic procedures, these leave the refilled tooth much more fragile and prone to fracture than natural teeth [5]. Even the widespread introduction of dental implants for complete tooth replacement has not come without problems, because the dental implant root is directly anchored to the alveolar bone, leading to the deficient cushioning of mastication impacts and the long-term development of conditions such as marginal bone loss and peri-implantitis [6,7]. Therefore, there is a clear demand for new biomaterials which allow not only for mechanical but also biological integration within restored dental tissues. It is to be expected that these kinds of bioactive materials will interact with the host cells and the oral environment to induce endogenous tissue regeneration and prevent future dental decay. Thus, bioactive materials are expected to be the cornerstone of next-generation dentistry.

In a previous Special Issue of Bioengineering, edited by Prof. Roy George (https://www.mdpi.com/journal/bioengineering/special_issues/dental_material; accessed on 5 December 2022), some interesting papers were published regarding the upgrading and functionalization of dental resin composites and pulp refilling materials with the incorporation of antibacterial compounds which would prevent secondary caries and root canal re-infections. Secondary caries is an overwhelming health problem that is responsible for the vast majority of failed tooth restorations [8]. These secondary infections are associated with the fact that currently employed dental adhesives and resin composites promote bacterial adhesion and growth to the restoration surface [9]. Moreover, these materials are prone to degradation over time, leading to the appearance of fractures and the need for serial interventions, which further weaken the teeth [10]. Some issues related to dental restoration failure could be addressed by incorporating contact-killing compounds such as Quaternary Ammonium [11] or metal oxide nanoparticles and nanotubes [12] in the resin composite, which would effectively inhibit the growth of biofilm-forming bacteria. These strategies have already been tested in some clinical pilot studies, like the one reported by Melo et al., where bis (2-methacryloyloxyethyl) dimethylammonium bromide (QADM) was copolymerized with dental composite resins to generate a wearable palatal device that could effectively reduce the growth of cariogenic bacteria [13]. Other strategies include the incorporation of nanoparticles of amorphous calcium phosphate (NACP), which results in the long-term release of calcium and phosphate ions that regenerate hydroxyapatite crystals, thus promoting natural tooth remineralization [14]. Finally, the incorporation of natural polyphenols in dental refilling materials is to be regarded as a highly innovative, safe, and low-cost strategy to upgrade dental restoration materials [15]. It has been shown that some polyphenolic compounds significantly enhance the adhesion of composite resins to dentin [16], a phenomenon that can be at least partly attributed to cross-linking with dentin collagen [15,17]. In addition, these natural polyphenols also have well-reported anti-inflammatory and anti-bacterial properties, which are also beneficial for the enhanced stability of dental restorations and better oral health with minimal side effects [15].

Overall, the current trend suggests that in the next decade, rapid growth in the development and clinical testing of bioactive upgraded dental re-filling materials will likely take place, driven in no small part by the strong interests of the dental industry in its pursuit to generate new products with advanced functionalities.

Endodontic procedures and root canal sealing with inert materials (e.g., gutta-percha) are currently the treatment of choice against irreversible dental pulpitis. The main problem associated with modern endodontics is that the tooth pulp is completely removed and deprived of its endogenous maintenance and mineralization capacity. In the absence of a functional dental pulp containing dentin-producing odontoblasts, the tooth is left with a much higher propensity to undergo fracture and secondary complications [5]. The issues associated with dental pulp removal could be completely prevented by replacing the currently used inert refilling materials with bioactive materials, which may also be functionalized with cells and/or growth factors, to promote the effective regeneration of the dental pulp tissue. These biomaterials should induce the migration of cells and blood vessels through the apical foramen to reconstruct the whole dental pulp in a process referred to as “Cell Homing” [18]. Dental pulp regeneration has been in the spotlight of the dental field ever since Nakashima and Iohara performed a successful clinical pilot study with sound results of pulp vitality recovery and regeneration after 24 weeks of transplantation of autologous patient-specific dental pulp stem cells (DPSCs) in 2017 [19]. However, stem cell-based treatments are costly and require cellular manipulation under good manufacturing practice (GMP) conditions, which are difficult and likely unrealistic to implement in a regular dental clinic. In recent years, however, different clinical trials in human patients have evaluated the safety and efficacy of cell-homing strategies to regenerate dental pulp, based on pulpectomized teeth refilling with collagen, platelet-rich plasma, and platelet-rich fibrin scaffolds, with particularly good results in the case of pulp regeneration on immature teeth [18]. Some human-derived scaffolds, such as those derived from autologous blood plasma, are known to release growth factors with osteogenic, angiogenic, and chemotactic activity [20,21,22], which would likely contribute to the recolonization of the refilled pulp tissue. Because of the large potential benefits associated with the implementation of cell-homing-based dental pulp regeneration therapies, this field of research is also bound to progress significantly over the next few decades.

Perhaps one of the most difficult remaining challenges in modern dentistry is the regeneration of a functional periodontal ligament (PDL) after the placement of a prosthetic dental implant. The PDL is a narrow strip of fibrous collagen-rich and highly vascularized connective tissue that connects the dental root with the surrounding alveolar bone, substantially cushioning mastication impacts. After a complete dental extraction, the empty alveolus is naturally refilled with compact bone, which is eventually used to screw the implant root pillar onto. However, because the implant stays bound in direct contact with the alveolar bone, the latter is inevitably subjected to a much higher mechanical load than it would be in normal teeth, usually causing marginal bone loss and risk of peri-implantitis in the long term [6]. The accumulation of dental plaque biofilms and the persistent inflammation associated with bone micro-fractures related to excessive mechanical load on the implant surface only accelerate this process [23]. Therefore, there is an unmet need for biomaterials that can regenerate a PDL-like tissue around dental implants to enhance their long-term durability. The main issue with conventional bioscaffolds like collagen or fibrin-related products is that they tend to favor mineralization and bone deposition on the implant surface [22,24,25]. Whereas these scaffolds constitute excellent alternatives for the reconstruction of periodontal bone defects [21,22], an effective PDL reconstruction strategy should also ideally include a biomaterial that is refractory to mineralization. Recently, we described a possible role for human Decellularized Adipose Tissue (hDAT) in this context, as we demonstrated that this biomaterial possessed a markedly lower capacity to be mineralized by osteogenic stem cells than other conventional scaffolds like collagen [26]. Could hDAT and other related biomaterials be used someday to surround prosthetic implant roots to attach them to the alveolar bone, to prevent dental implant ankylosis? Arguably, we are only recently taking the first steps toward the generation of better biomimetic dental implants, which may incorporate not only the replacement tooth but also its associated PDL. Such a development would constitute a real revolution in dental implantology.

We are witnessing ever-growing research activity in the fields of tissue engineering and bioactive materials for dentistry applications. Contrary to last-generation dental materials, which were largely chosen because of their inert nature and lack of adverse reactions, next-generation dental materials will be expected to exert true biological effects in the surrounding oral and dental tissues to promote better and more functional integration.
